# Association between frequency of breakfast consumption and cardiometabolic risk in Peruvian university teachers, 2019–2020

**DOI:** 10.3389/fnut.2023.1238223

**Published:** 2023-07-28

**Authors:** Jacksaint Saintila, Laura E. Baquedano-Santana, Yaquelin E. Calizaya-Milla, Cristian Ramos-Vera, Antonio Serpa Barrientos, Renzo F. Carranza-Esteban

**Affiliations:** ^1^Escuela de Medicina Humana, Universidad Peruana Unión, Lima, Peru; ^2^Research and Development Laboratories, Faculty of Sciences and Philosophy, Universidad Peruana Cayetano Heredia, Lima, Peru; ^3^Research Group for Nutrition and Lifestyle, Universidad Peruana Unión, Lima, Peru; ^4^Área de Investigación, Universidad Cesar Vallejo (UCV), Lima, Peru; ^5^Departamento Psicología, Universidad Nacional Mayor de San Marcos, Lima, Peru; ^6^Grupo de Investigación Avances en Investigación Psicológica, Facultad de Ciencias de la Salud, Universidad San Ignacio de Loyola, Lima, Peru

**Keywords:** breakfast eating, obesity, glucose, triglycerides, LDL cholesterol, dyslipidemia, hypertension

## Abstract

**Background:**

Although the relationship between health status and dietary intake has been extensively studied in the general population, there is a lack of research that has specifically examined the association between frequency of breakfast consumption and cardiometabolic risk in university teachers.

**Objective:**

To determine the association between the frequency of breakfast consumption and cardiometabolic risk in university teachers.

**Methods:**

A cross-sectional study was conducted in 176 teachers from a private university located in the eastern region of Lima, Peru (*M*_age_: 37.0 years; SD: 0.8, range: 24–59 years). The study was conducted during the period from December 2019 to February 2020. Information was collected on anthropometric and biochemical parameters and frequency of breakfast consumption. Multinomial logistic regression models were used to explore the association between frequency of breakfast with sociodemographic, anthropometric, and biochemical variables.

**Results:**

The highest prevalence of excess body weight (44.4%) was observed in those who consumed breakfast 0 to 2 days/week, but without statistical differences. Those who reported Low-density lipoprotein cholesterol (LDL-C) < 160 mg/dL were 77% less likely to fall into the 3–5 day/week breakfast frequency category than those who reported a regular frequency of breakfast (6 to 7 days/week) (Adjusted OR = 0.23, 95% CI 0.08 to 0.73; *p* < 0.05). In addition, teachers who reported a breakfast frequency of 3 to 5 days/week were 83% more likely to have a glucose concentration < 110 mg/dL compared to those who consumed breakfast of 6 to 7 days/week (Adjusted OR = 0.17, 95% CI 0.04 to 0.75; *p* < 0.05).

**Conclusion:**

Skipping breakfast for an extended period of time can have detrimental effects on cardiometabolic health. Promoting the benefits of breakfast could be a health message of great public health interest.

## Introduction

The prevalence of noncommunicable diseases continues to increase at an exponential rate worldwide (1). In particular, cardiometabolic diseases, such as diabetes and hypertension, and respiratory diseases, as well as cancer were responsible for the deaths of 41 million people worldwide annually, representing 74% of all deaths worldwide, 77% of which occurred in low- and middle-income countries (2). In Peru, in 2021, 17.2% of the Peruvian population over 15 years of age had high blood pressure, 4.9% had type 2 diabetes mellitus, and 25.8% were obese (3). In particular, obesity registered an increase of 1.2 percentage points over the previous year (4). Health risk behaviors, such as smoking (5), lack of physical activity (6), excessive alcohol consumption (7), and an unhealthy diet characterized by foods high in saturated fats, free sugars, sodium, and low in fiber (8–10), increase the risk of death due to cardiometabolic diseases.

On the other hand, generally inadequate food consumption practices, such as lack of regularity in the frequency of breakfast consumption, have emerged as significant contributing factors in cardiovascular and metabolic diseases (11–13). Particularly, modification of breakfast patterns may be important, because skipping breakfast was associated with an increased risk of all-cause mortality (11). In fact, a systematic review provided evidence that skipping breakfast may lead to an increased risk of developing overweight and obesity (13). In addition, previous studies have shown that skipping breakfast is associated with elevated levels of plasma lipoproteins and fasting blood glucose (14, 15). Similarly, it has been observed that skipping breakfast is related to an inadequate intake of essential nutrients (16); by skipping this important meal, it is more likely to exclude the intake of key nutrients that are necessary for the optimal functioning of the body, which can lead to obesity. Importantly, the mechanism linking breakfast skipping with obesity may be related to negative effects on circadian rhythm, which may lead to irregularity in metabolism and, therefore, consequences in weight control (17). In fact, reducing the frequency of consumption of other meals, such as lunch combined with skipping breakfast are constituted as factors of a less healthy lifestyle and are associated with weight gain (16).

In contrast, studies have shown that eating breakfast regularly is associated with a lower body mass index (BMI) in both adults and adolescents, and may act as a preventive measure to avoid the onset of obesity in the future (12). In addition, research based on the observation of breakfast frequency suggests that there is an inverse (i.e., protective) relationship between eating breakfast frequently and the risk of developing cardiometabolic diseases, such as type 2 diabetes and obesity, demonstrating positive effects on blood glucose levels (18). Breakfast is considered the most important meal of the day. Therefore, international recommendations regarding breakfast are aligned in stating that its daily consumption is recommended to provide an adequate intake of macronutrients and micronutrients, as well as to maintain a healthy body weight (19, 20).

Considering the enormous medical, financial, and social burden of cardiometabolic diseases, it is necessary to focus attention not only on traditional populations, such as health care providers, public officials, and bankers, but also on university teachers because it is likely that they may be exposed to an increased risk of cardiometabolic diseases due to lifestyle changes and ongoing urbanization (21). However, although the link between health status and dietary intake has been the subject of scientific scrutiny in recent years in the general population (22, 23). However, there are few studies that have evaluated the association between the frequency of breakfast consumption and cardiometabolic risk in university teachers. Therefore, this study aimed to determine the association between the frequency of breakfast consumption and cardiometabolic risk through BMI, waist circumference (WC), low-density lipoprotein cholesterol (LDL-C), high-density lipoprotein cholesterol (HDL-C), and triglycerides (TG) in university teachers.

It is expected that this study will provide evidence on the relationship between breakfast consumption and cardiometabolic risk factors in this specific group, filling the gap and providing information relevant to the health and well-being of university teachers. Furthermore, by considering multiple cardiometabolic risk parameters, such as BMI, WC, LDL-C, HDL-C, and TGs, it is hoped that the findings may provide a more complete picture of the possible effects of breakfast consumption on cardiovascular and metabolic health in this population. This is particularly important because it would favor the implementation of nutritional education programs and strategies to adopt healthy lifestyle, more specifically, healthy breakfast habits, which can help reduce the risk of cardiometabolic diseases in university teachers.

## Materials and methods

### Design and participants

A cross-sectional study was conducted between December 2019 and February 2020, in which a pre-structured survey was applied to evaluate anthropometric parameters (weight, height, BMI, and WC), biochemical profiles (HDL-C, LDL-C, TG, and glucose), and breakfast frequency in a group of teachers from a private university located in the eastern region of Lima, Peru. A non-probabilistic convenience sampling approach was used, because the sample was based on the specific criteria of the researchers, taking into account the ease of access of the participants (24). We excluded those who did not sign the informed consent, those who underwent medical treatment, and those with chronic noncommunicable diseases such as diabetes, cardiovascular disease, dyslipidemia, and arterial hypertension. Additionally, a total of 11 records were excluded because they did not correctly complete all items related to sociodemographic data. The final sample was 176 university teachers.

### Procedures and ethical aspects

In the first instance, data collection was carried out after the project had been evaluated and received approval from the Research Ethics Committee of the Faculty of Health Sciences of the Universidad Peruana Unión (Registration and reference number: N°00124-2019/UPeU/FCS/CIISA). Second, permission was obtained from the university to survey and evaluate the teachers. Third, before applying the survey, the purpose of the study was explained to each teacher. Written informed consent was obtained from all participants. In addition, all procedures carried out in this study adhered to the ethical standards established in the Declaration of Helsinki. Finally, the confidentiality of the data and the voluntary and anonymous nature of participation were respected.

### Variables

#### BMI

The weight and height of the participants were assessed using a calibrated SECA 700 mechanical column scale (SECA®, Hamburg, Germany). This scale has a capacity of 220 kg and a measuring range of 60 to 200 cm. Then, the BMI was calculated using the formula weight divided by height squared (kg/m^2^). This measure evaluates the relationship between the weight and height of each participant and is used to classify the nutritional status according to the criteria established by the Peruvian Ministry of Health in the Technical Guide for the Anthropometric Nutritional Assessment of Adults (25). Participants were classified into two groups as follows: BMI <25 and BMI ≥25 (26). In Peru and according to WHO recommendations, a BMI ≥25 is considered overweight (25).

#### CMR

CMR was determined through the measurement of WC using a Cescorf (Cescorf Equipamentos Para Esporte Ltda - Epp, Brazil) self-retracting metallic steel tape measure. The study used the CMR criterion, measured through the WC, established in the Peruvian Technical Guide for the anthropometric nutritional assessment of adults (25). According to this criterion, it was considered that CMR exists if the WC is equal to or greater than 94 cm in men and equal to or greater than 80 cm in women (25).

#### Biochemical data

Blood collection (5 mL) was performed during the first two hours of the morning, after 12 h of fasting according to standard procedures for blood samples (27). *Wiener lab commercial Colestat enzymatic AA* kits were used to determine the lipid profile (28). Standardized techniques based on enzymatic and colorimetric methods were employed, using spectrophotometry, following the manufacturer’s recommendations. In addition, measurements were performed using a semi-automatic biochemical analyzer. These methods guarantee accuracy and reliability in the determination of the different components of the lipid profile, providing relevant information on the cardiovascular health of the participants (28). Subsequently, glycaemia was measured manually using colorimetric enzymatic methods (29). Participants were classified according to their lipid profile as follows: high LDL-C levels (LDL-C ≥ 160 mg/dL), low HDL-C levels (HDL-C < 40 mg/dL in men and < 50 mg/dL in women), and presence of hypertriglyceridemia (TG ≥ 200 mg/dL) were considered (30). In addition, glycaemia was considered when the fasting glucose concentration was <110 mg/dL (30). The extraction and processing of the serum was carried out by a certified and trained Medical Technologist at the Microbiology Laboratory of the Universidad Peruana Unión.

#### Frequency of breakfast consumption

A standard questionnaire previously used in other investigations was used to assess the frequency of breakfast consumption (31). Each participant was asked to answer the question “*How often do you eat breakfast in a week?*.” Participants provided a numerical response from 0 to 7 for this question. Breakfast frequency was categorized into three groups: “rarely or never (0–2 days),” “some days (3–5 days),” and “regularly (6–7 days)” (12).

#### Sociodemographic data

Data were collected on sex (male, female); age; place of origin (coast, highlands, and jungle); marital status (married and single); degree of study (post-graduate and undergraduate), and dietary pattern (vegetarian and non-vegetarian).

### Statistical analysis

Descriptive exploration of the data was carried out using the Kruskal Wallis test for continuous variables expressed as mean value (*M*) and standard deviation (SD) and the Chi-square (*χ*2) statistic for categorical variables (reported as absolute frequencies and percentage). Multinomial logistic regression models were used to explore the relationship between categories of frequency of breakfast consumption with sociodemographic, anthropometric, and biochemical variables. Crude and Adjusted Odds ratios (OR) with confidence intervals (95% CI) were calculated. Model was adjusted for age, gender, place of origin, marital status, degree of education, dietary pattern, and BMI factors. We considered a value of p of less than 0.05 as significant. Analyses were performed with SPSS version 25 statistical software (SPSS Inc., Chicago, IL, USA).

## Results

A total of 176 university teachers participated, of which 63.6% were male and had a mean age of 37 years (SD _age_ = 0.8). Approximately 46% were from the coastal areas of the country. More than half were married (51.7%), had a postgraduate degree (58.5%), and were non-vegetarians (60.2%). Teachers who consumed breakfast 0 to 2 days/week were mostly married (59.3%) and with a graduate degree (63.0%). The highest proportion of those who consumed breakfast 6 to 7 days/week were women (64.2%) and non-vegetarians (63.2%) (Table 1).

**Table 1:** Sociodemographic factors and dietary patterns according to frequency of breakfast consumption in university teachers (*N* = 176).

Variables		Total	Frequency of breakfast consumption			
		*n* (%)	0–2 D/W	3–5 D/W	6–7 D/W	*X*^2^‡
			*n* (%)			
Age† (*M* ± SD)		37 ± 0.8 (176)	35.9 ± 1.8 (27)	34.5 ± 1.4 (43)	38.3 ± 1.0 (106)	0.161
Gender						1.043
	Female	112 (63.6)	15 (55.6)	29 (67.4)	68 (64.2)	
	Male	64 (36.4)	12 (44.4)	14 (32.6)	38 (35.8)	
Place of origin						3.786
	Coast	81 (46.0)	12 (44.4)	20 (46.5)	49 (46.2)	
	Jungle	51 (29.0)	10 (37.0)	15 (34.9)	26 (24.5)	
	Sierra	44 (25.0)	5 (18.5)	8 (18.6)	31 (29.2)	
Marital status						2.468
	Single	85 (48.3)	11 (40.7)	25 (58.1)	49 (46.2)	
	Married	91 (51.7)	16 (59.3)	18 (41.9)	57 (53.8)	
Degree of education						0.705
	Graduate	73 (41.5)	10 (37.0)	20 (46.5)	43 (40.6)	
	Postgraduate	103 (58.5)	17 (63.0)	23 (53.5)	63 (59.4)	
Dietary pattern						1.219
	Vegetarian	70 (39.8)	11 (40.7)	20 (46.5)	39 (36.8)	
	Non-vegetarian	106 (60.2)	16 (59.3)	23 (53.5)	67 (63.2)	

‡Overall group differences, based on Kruskal Wallis for continuous variables and Chi-square (*X*^2^) statistics for categorical variables; *p < 0.05 (significant). †Age in years.

M, Mean; SD, standard deviation; D/W, Days/Week.

In this study sample, most of the teachers did not present CMR, had LDL-C, TG, and glucose concentration at normal levels; however, they had an HDL-C level below normal ranges (<40 mg/dL males, <50 mg/dL females). The highest prevalence of excess body weight (44.4%) was observed in those teachers who consumed breakfast 0 to 2 days/week, but without statistical differences. Significant differences were observed in the distributions of LDL-C (*p* = 0.024) and glucose (*p* = 0.038) levels in the three groups of teachers (Table 2).

**Table 2:** Cardiovascular risk factors according to the frequency of breakfast consumption in university teachers.

Variables		Total	Frequency of breakfast consumption			
		*n* (%)	0–2 D/W	3–5 D/W	6–7 D/W	*X*^2^‡
			*n* (%)			
BMI						1.111
	<25	113 (64.2)	15 (55.6)	29 (67.4)	69 (65.1)	
	≥25	63 (35.8)	12 (44.4)	14 (32.6)	37 (34.9)	
WC						1.697
	Non CMR	121 (68.8)	18 (66.7)	33 (76.7)	70 (66.0)	
	CMR	55 (31.3)	9 (33.3)	10 (23.3)	36 (34.0)	
LDL-C						7.446*
	<160 mg/dL	158 (89.8)	26 (96.3)	34 (79.1)	98 (92.5)	
	≥160 mg/dL	18 (10.2)	1 (3.7)	9 (20.9)	8 (7.5)	
HDL-C						0.973
	≥40 mg/dL M, ≥50 mg/dL F	85 (48.3)	14 (51.9)	18 (41.9)	53 (50.0)	
	<40 mg/dL M,<50 mg/dL F	91 (51.7)	13 (48.1)	25 (58.1)	53 (50.0)	
TG						0.838
	<200 mg/dL	164 (93.2)	26 (96.3)	39 (90.7)	99 (93.4)	
	≥200 mg/dL	12 (6.8)	1 (3.7)	4 (9.3)	7 (6.6)	
Glucose						6.532*
	<110 mg/dL	165 (93.8)	25 (92.6)	37 (86.0)	103 (97.2)	
	≥110 mg/dL	11 (6.3)	2 (7.4)	6 (14.0)	3 (2.8)	

‡Overall group differences, based on Chi-square (*X*^2^) statistics for categorical variables; *p < 0.05 (significant).

D/W, Days/Week; BMI, Body mass index; WC, Waist circumference; CMR, cardiometabolic risk; LDL-C, low-density lipoprotein cholesterol; HDL-C, high-density lipoprotein cholesterol; TG, triglyceride; M, males; F, females.

The findings of the multinomial logistic regression analysis showed that there was no statistically significant relationship between sociodemographic factors, dietary pattern, and BMI with the frequency of breakfast consumption (see Table 3).

**Table 3:** Odds ratios (OR) and 95% confidence intervals (CI) of sociodemographic factors and dietary patterns in relation to frequency of breakfast consumption.

Frequency of breakfast consumption (a)			*B*	Crude OR (95% CI)	Adjusted OR (95% CI)
0–2 D/W	Age (years)		−0.036	0.98 (0.94–1.02)	0.96 (0.91–1.02)
	Gender	Female	−0.428	0.70 (0.30–1.65)	0.65 (0.26–1.66)
		Male		–	–
	Place of origin	Coast	0.400	1.52 (0.49–4.73)	1.49 (0.46–4.88)
		Jungle	0.778	2.39 (0.72–7.87)	2.18 (0.59–8.06)
		Sierra		–	–
	Marital status	Single	−0.298	0.80 (0.34–1.89)	0.74 (0.25–2.19)
		Married		–	–
	Degree of education	Graduate	−0.269	0.86 (0.36–2.06)	0.76 (0.29–2.02)
		Postgraduate		–	–
	Dietary pattern	Vegetarian	0.319	1.18 (0.50–2.80)	1.38 (0.50–3.83)
		Non-vegetarian		–	–
3–5 D/W	Age (years)		−0.036	0.96 (0.93–1.00)*	0.97 (0.92–1.01)
	Gender	Female	0.060	1.16 (0.55–2.45)	1.06 (0.47–2.42)
		Male		–	–
	Place of origin	Coast	0.331	1.58 (0.62–4.03)	1.39 (0.53–3.67)
		Jungle	0.585	2.24 (0.82–6.10)	1.79 (0.60–5.37)
		Sierra		–	-
	Marital status	Single	0.348	1.62 (0.79–3.31)	1.42 (0.57–3.53)
		Married		–	–
	Degree of education	Graduate	0.484	1.27 (0.62–2.60)	1.62 (0.74–3.57)
		Postgraduate		–	–
	Dietary pattern	Vegetarian	0.509	1.49 (0.73–3.06)	1.66 (0.71–3.91)
		Non-vegetarian		–	–

^a^The reference category is 6–7 Days/Week.

Multinomial logistic regression analysis was used in statistical analysis. *p < 0.05 (significant). Model adjusted for age, gender, place of origin, marital status, degree of education, dietary pattern, and BMI factors.

OR, odds ratio; CI, confidence interval; B, Regression coefficient; D/W, Days/Week.

Table 4 showed that participants who reported Low-density lipoprotein cholesterol (LDL-C) < 160 mg/dL were 77% less likely to fall into the 3–5 day/week breakfast frequency category than those who reported a regular frequency of breakfast (6 to 7 days/week) (β = −1.456; Adjusted OR = 0.23, 95% CI 0.08 to 0.73; *p* < 0.05). In relation to glucose concentration, teachers who reported a breakfast frequency of 3 to 5 days/week were 83% more likely to have a glucose concentration < 110 mg/dL compared to those who consumed breakfast of 6 to 7 days/week (*β* = −1.804; Adjusted OR = 0.17, 95% CI 0.04–0.75; *p* < 0.05). In addition, participants who had LDL-C < 160 mg/dL and Triglyceride (TG) < 200 mg/dL had 1.60 and 2.91 times the odds of belonging to the 0 to 2 days/week breakfast frequency category compared to those who consumed breakfast 6 to 7 days/week, however, no significant difference was observed (*p* = 0.673 and *p* = 0.347, respectively).

**Table 4:** Odds ratios (OR) and 95% confidence intervals (CI) of cardiovascular risk factors in relation to frequency of breakfast consumption.

Frequency of breakfast consumption (a)			*B*	Crude OR (95% CI)	Adjusted OR (95% CI)
0–2 D/W	BMI	<25	−0.777	0.67 (0.28–1.58)	0.46 (0.17–1.27)
		≥25		–	–
	WC	Non CMR	0.083	1.03 (0.42–2.52)	1.09 (0.34–3.51)
		CMR		–	–
	LDL-C	<160 mg/dL	0.469	2.12 (0.25–17.74)	1.60 (0.18–14.12)
		≥160 mg/dL		–	–
	HDL-C	≥40 mg/dL M, ≥50 mg/dL F	−0.045	1.08 (0.46–2.51)	0.96 (0.39–2.33)
		<40 mg/dL M, <50 mg/dL F			–
	TG	<200 mg/dL	1.067	1.84 (0.22–15.62)	2.91 (0.31–26.85)
		≥200 mg/dL		–	–
	Glucose	<110 mg/dL	−1.097	0.36 (0.06–2.30)	0.33 (0.05–2.30)
		≥110 mg/dL		–	–
3–5 D/W	BMI	<25	−0.359	1.11 (0.52–2.36)	0.70 (0.29–1.71)
		≥25		–	–
	WC	Non CMR	0.469	1.70 (0.75–3.83)	1.60 (0.56–4.52)
		CMR		–	–
	LDL-C	< 160 mg/dL	−1.456	0.31 (0.11–0.86)*	0.23 (0.08–0.73)*
		≥ 160 mg/dL		–	–
	HDL-C	≥40 mg/dL M, ≥50 mg/dL F	−0.399	0.72 (0.35–1.47)	0.67 (0.31–1.45)
		<40 mg/dL M, <50 mg/dL F			–
	TG	<200 mg/dL	−0.144	0.69 (0.19–2.49)	0.87 (0.22–3.37)
		≥200 mg/dL		–	–
	Glucose	<110 mg/dL	−1.804	0.18 (0.04–0.76)*	0.17 (0.04–0.75)*
		≥110 mg/dL		–	–

^a^The reference category is 6–7 Days/Week.

Multinomial logistic regression analysis was used in statistical analysis. *p < 0.05 (significant). Model adjusted for age, gender, place of origin, marital status, degree of education, dietary pattern, and BMI factors.

OR: odds ratio; CI: confidence interval; B, Regression coefficient. D/W, Days/Week; BMI, Body mass index; WC, Waist circumference; CMR, cardiometabolic risk; LDL-C, low-density lipoprotein cholesterol; HDL-C, high-density lipoprotein cholesterol; TG, triglyceride; M, males; F, females.

## Discussion

In this cross-sectional study, we determined the association between cardiometabolic risk and the frequency of breakfast consumption in a group of university teachers. We found that the highest prevalence of excess body weight was observed in those who consumed breakfast rarely or never (0 to 2 days/week), although there were no statistical differences. Likewise, it was evidenced that those who reported an LDL-C lower than 160 mg/dL were 77% less likely to have breakfast some days (3 to 5 days/week) than those who reported having breakfast regularly (6 to 7 days/week), that is, a significant reduction in the probabilities of having a frequency of breakfast consumption of 3 to 5 days a week. These results suggest that those with lower LDL-C levels are less likely to eat breakfast at that specific frequency (3 to 5 days a week), compared to those with higher LDL-C levels. Similarly, teachers who reported a breakfast frequency of 3 to 5 days/week were 83% more likely to have a glucose concentration < 110 mg/dL compared to those who consumed breakfast of 6 to 7 days/week.

Obesity is one of the most worrisome public health problems in the world. In the present study, although there was no significant difference, teachers who reported a frequency of breakfast 0 to 2 days/week were found to have a higher prevalence of excess body weight. Evidence suggests that skipping breakfast or having an infrequent breakfast has been associated with an increased risk of developing obesity. For example, a systematic review has compiled evidence suggesting that skipping breakfast may be associated with weight gain and the development of overweight and obesity (13). In fact, it has been observed that decreasing the frequency of consumption of other meals, such as lunch, in combination with skipping breakfast, is associated with a less healthy lifestyle and an increased risk of weight gain (16). In contrast, a recent study examining the association between breakfast frequency and body size in elementary school teachers revealed that regular breakfast consumption (6–7 days/week) had a significant effect on obesity. This suggests that regular breakfast is a protective factor in this study; they also reported that teachers who rarely ate breakfast were more likely to suffer from obesity, according to the odds ratio calculated (12).

One possible justification for these findings is that breakfast provides energy and important nutrients that help regulate metabolism and control appetite throughout the day (32–34). Additionally, it has been observed that people who consume breakfast regularly tend to have a healthier diet and healthier eating patterns in general; they are also less likely to feel hungry for snacks during the day than people who skip breakfast, which may reduce the risk of excess body weight (35, 36). On the other hand, it is relevant to highlight that the underlying mechanism that connects lack of breakfast with obesity could be associated with detrimental effects on the circadian rhythm, which could lead to an alteration in metabolism and, consequently, have implications for body weight control (17). It is important to note that the relationship between breakfast skipping and obesity is complex and may be influenced by other underlying factors such as stress, lack of time, or lack of knowledge about healthy breakfast options. Therefore, it is essential to emphasize that addressing the relationship between breakfast frequency and obesity requires a comprehensive approach that includes education, habit change, and the creation of enabling environments to encourage regular and healthy breakfast among university teachers.

On the other hand, was evidenced that teachers who reported an LDL-C lower than 160 mg/dL were 77% less likely to have breakfast some days (3–5 days/week) than those who reported having breakfast regularly (6–7 days/week). In addition, an inverse relationship between the frequency of breakfast consumption 3–5 days/week and LDL-C concentration was evidenced. Thus, the results suggest that eating breakfast 0 to 2 days per week or 3 to 5 days per week may be associated with a lower likelihood of maintaining LDL-C levels below 160 mg/dL compared with eating breakfast 6 to 7 days a week, as the frequency of breakfast increases in that specific range, a decrease in LDL-C levels is observed. In other words, those individuals who regularly consume breakfast 6 to 7 days/week tend to have lower levels of LDL-C compared to those who eat breakfast less frequently. This association suggests that regular consumption of breakfast may have a beneficial effect on the regulation of LDL-C. It is important to mention that the relationship between LDL-C levels and frequency of breakfast consumption is not fully established in the scientific literature. However, some studies suggest a possible association between frequent breakfast consumption and more favorable lipid profiles, including adequate LDL-C levels. In that same order, findings from one study have shown that those participants who skip breakfast show a worse lipid profile compared to those who consume breakfast (37). Similarly, another study concluded that participants who skipped breakfast during both childhood and adulthood had higher levels of LDL-C compared to those who ate breakfast in both periods (38). Similarly, a study in adolescents reported an association between skipping breakfast and higher LDL-C concentrations (39). This underlines the importance of promoting a regular and balanced consumption of breakfast as part of a healthy and balanced diet.

Finally, in relation to glucose concentration, it was found that teachers who reported a breakfast frequency of 3–5 days/week were 83% more likely to have a glucose concentration < 110 mg/dL compared to those who consumed breakfast of 6 to 7 days/week. Similarly, a negative correlation was observed between a frequency of breakfast consumption 3–5 days/week and glucose concentration. Thus, multinomial logistic regression results suggest that eating breakfast 0 to 2 days/week or 3 to 5 days/ week may be associated with a lower probability of maintaining glucose levels below 110 mg/dL compared with eating breakfast 6 to 7 days/week. Our findings are similar with the results of a study that found fasting glucose was slightly higher in those who skipped breakfast as adults than in those who consumed breakfast (38). Additionally, these results are consistent with previous research that has reported an association between skipping breakfast and higher blood glucose levels, as well as a lower likelihood of maintaining good glycemic control (14, 15). It is possible that regular breakfast eaters may have a lower intake of foods high in saturated and trans fats and other calorie-dense foods that can raise LDL-C and blood glucose levels. Moreover, by eating breakfast regularly, it is more likely that more nutritious foods, such as fruits, vegetables, whole grains, and lean proteins, will be consumed, which can contribute to maintaining lower LDL-C levels and glucose concentrations. The understanding of these associations can favor decision-making on nutrition and the adoption of habits that favor better glycemic control and lipid profile, which can reduce the risk of cardiometabolic diseases in university teachers.

### Limitations

The findings of this research should be interpreted considering several limitations. First, the study was based on cross-sectional data rather than longitudinal data, it was not possible to establish a conclusive causal relationship between the frequency of breakfast consumption and cardiometabolic risk. Secondly, the results and conclusions of this study should be interpreted with caution, since the sample used was not randomly selected and a non-probabilistic selection method was used. This limits the generalizability of the findings to the general population. Third, it is important to mention that this study did not take into account the use of a frequency of consumption questionnaire to assess breakfast consumption. In addition, we did not evaluate other confounding factors, such as physical activity and income. These factors may have a significant impact not only on breakfast consumption but also on cardiometabolic risk. Therefore, it is suggested that future research should consider the inclusion of these variables to obtain a more complete understanding of the relationship between breakfast and cardiometabolic risk. In the study, hip circumference assessment was not performed, which prevented the analysis of the relationship between waist and hip measurement, known as waist-to-hip ratio. In addition, it is important to mention that the anthropometric indicators were measured only once, however, in some cases, it is recommended to perform two or three repetitions to obtain more accurate measurements (40).

### Implications for public health and future perspectives

Despite these limitations, we believe that understanding the association between the frequency of breakfast consumption and cardiometabolic risk is of great public health relevance because of its potential impact on the health and work performance of university teachers. Evidence suggests that skipping or irregular breakfast consumption is associated with an increased risk of developing cardiovascular and metabolic diseases, such as hypertension, dyslipidemia, and type 2 diabetes mellitus (41, 42). Therefore, promoting the regular and healthy consumption of breakfast can bring significant health benefits to university teachers. In addition, proper breakfast provides essential nutrients, helps regulate appetite, control weight, improve glucose and lipid metabolism, and can have positive effects on blood pressure (33, 34). In this sense, public health interventions and programs that promote the importance of breakfast and provide strategies to adopt healthy breakfast habits can help reduce the risk of cardiometabolic diseases and promote healthier lifestyles in the population, more specifically in university teachers. Finally, it is important to educate teachers and the general population about the importance of a balanced diet and proper meal planning, including a healthy breakfast, as part of a comprehensive strategy for the prevention and control of cardiometabolic diseases.

Additionally, longitudinal studies involving a larger sample of participants are suggested. This would increase the validity of the results and provide a better understanding of the associations between the frequency of breakfast consumption and health outcomes over time. The inclusion of long-term follow-ups would also provide more robust information on the long-term implications of skipping breakfast on health and well-being. In addition, qualitative research is suggested to gain a deeper understanding of the underlying factors and motivations behind breakfast skipping and its relationship with cardiometabolic risk. These investigations could involve interviews, focus groups, or case studies to explore people’s perceptions, beliefs, and behaviors regarding breakfast and its impact on health. These qualitative approaches can provide a more complete and contextualized view of the factors influencing breakfast choices and provide valuable information for the development of education programs with an emphasis on prevention.

## Conclusion

The results of this cross-sectional study showed that the highest prevalence of excess body weight was observed in those who consumed breakfast rarely or never (0 to 2 days/week), although there were no statistical differences. Moreover, it was found that teachers who reported an LDL-C lower than 160 mg/dL were 77% less likely to have breakfast some days (3–5 days/week) than those who reported having breakfast regularly (6–7 days/week). Similarly, those who reported a glucose concentration higher than 110 mg/dL breakfast frequency of 3–5 days/week were 83% more likely to have a glucose concentration ≥ 110 mg/dL compared to those who consumed regularly (6–7 days/week). Considering the significant impact that skipping breakfast can have on health, further efforts are needed to understand in greater depth the factors associated with this practice. This will allow the implementation of more effective preventive and therapeutic interventions to promote healthy eating habits, especially regarding breakfast.

## Data availability statement

The raw data supporting the conclusions of this article will be made available by the authors, without undue reservation.

## Ethics statement

The studies involving human participants were reviewed and approved by Research Ethics Committee of the Faculty of Health Sciences of the Universidad Peruana Unión (Registration and reference number: N°00124-2019/UPeU/FCS/CIISA). The patients/participants provided their written informed consent to participate in this study.

## Author contributions

JS was in charge of the protocol as the principal investigator. LB-S and YC-M collaborated in the survey design. LB-S and CR-V were in charge of data analysis. JS wrote the first draft of the manuscript. AB and RC-E contributed to the research and writing of the manuscript. All authors reviewed and approved the final version of the manuscript.

## Funding

The study was financed by the Universidad Peruana Unión (Resolución N° 2556-2021/UPeU-CU).

## Conflict of interest

The authors declare that the research was conducted in the absence of any commercial or financial relationships that could be construed as a potential conflict of interest.

## Publisher’s note

All claims expressed in this article are solely those of the authors and do not necessarily represent those of their affiliated organizations, or those of the publisher, the editors and the reviewers. Any product that may be evaluated in this article, or claim that may be made by its manufacturer, is not guaranteed or endorsed by the publisher.

## References

[1] Pengpid S Peltzer K. Prevalence and correlates of multiple behavioural risk factors of non-communicable diseases among university students from 24 countries. J Public Health. (2021) 43:857–66. doi: 10.1093/pubmed/fdaa138, PMID: 34918087

[2] World Health Organization (WHO). Noncommunicable diseases [Internet]. (2022). Available at: https://www.who.int/news-room/fact-sheets/detail/noncommunicable-diseases (Accessed April 30, 2023).

[3] INEI. Perú: Enfermedades No Transmisibles y Transmisibles, 2021 - Informes y publicaciones - Instituto Nacional de Estadística e Informática - Gobierno del Perú [Internet]. Lima. (2022). Available at: https://www.gob.pe/institucion/inei/informes-publicaciones/2983123-peru-enfermedades-no-transmisibles-y-transmisibles-2021 (Accessed September 30, 2023).

[4] INEI. El 39,9% de peruanos de 15 y más años de edad tiene al menos una comorbilidad [Internet]. (2020). Available at: https://www.inei.gob.pe/prensa/noticias/el-399-de-peruanos-de-15-y-mas-anos-de-edad-tiene-al-menos-una-comorbilidad-12903/

[5] Mishra VK Srivastava S Muhammad T Murthy PV. Relationship between tobacco use, alcohol consumption and non-communicable diseases among women in India: evidence from National Family Health Survey-2015-16. BMC Public Health. (2022) 22:713. doi: 10.1186/s12889-022-13191-z, PMID: 35410193 PMC8996590

[6] Jalayondeja C Jalayondeja W Mekhora K Bhuanantanondh P Dusadi-Isariyavong A Upiriyasakul R. Break in sedentary behavior reduces the risk of noncommunicable diseases and cardiometabolic risk factors among workers in a petroleum company. Int J Environ Res Public Health. (2017) 14:501. doi: 10.3390/ijerph14050501, PMID: 28486414 PMC5451952

[7] Ramos-Vera C Serpa Barrientos A Calizaya-Milla YE Carvajal Guillen C Saintila J. Consumption of alcoholic beverages associated with physical health status in adults: secondary analysis of the health information national trends survey data. J Prim Care Community Health. (2022) 13:215013192110662. doi: 10.1177/21501319211066205 PMC8744155 34991399

[8] Lloyd-Jones DM Hong Y Labarthe D Mozaffarian D Appel LJ Van Horn L. Defining and setting national goals for cardiovascular health promotion and disease reduction. Circulation. (2010) 121:586–613. doi: 10.1161/CIRCULATIONAHA.109.192703, PMID: 20089546

[9] Melaku YA Renzaho A Gill TK Taylor AW Dal Grande E de Courten B. Burden and trend of diet-related non-communicable diseases in Australia and comparison with 34 OECD countries, 1990–2015: findings from the Global Burden of Disease Study 2015. Eur J Nutr. (2019) 58:1299–313. doi: 10.1007/s00394-018-1656-7, PMID: 29516222

[10] Banda-Ccana DE Infantes-Ruiz VH Calizaya-Milla YE Saintila J. Diet and risk of mental illness in Peruvian adults, cross-sectional study [Dieta y riesgo de enfermedades mentales en adultos peruanos, estudio transversal]. Arch Latinoam Nutr. (2021) 71:199–207. doi: 10.37527/2021.71.3.004

[11] Helo D Appiah L Bhende KM Byrd TL Appiah D. The association of skipping breakfast with cancer-related and all-cause mortality in a national cohort of United States adults. Cancer Causes Control. (2021) 32:505–13. doi: 10.1007/s10552-021-01401-9, PMID: 33590466

[12] Uvacsek M Simkó G Boda-Ujlaky J Kneffel Z. Frequency of breakfast eating and obesity prevalence in primary school teachers. Int J Environ Res Public Health. (2022) 19:5331. doi: 10.3390/ijerph19095331, PMID: 35564728 PMC9105426

[13] Wicherski J Schlesinger S Fischer F. Association between breakfast skipping and body weight—a systematic review and Meta-analysis of observational longitudinal studies. Nutrients. (2021) 13:272. doi: 10.3390/nu13010272, PMID: 33477881 PMC7832891

[14] Ogata H Kayaba M Tanaka Y Yajima K Iwayama K Ando A. Effect of skipping breakfast for 6 days on energy metabolism and diurnal rhythm of blood glucose in young healthy Japanese males. Am J Clin Nutr. (2019) 110:41–52. doi: 10.1093/ajcn/nqy346, PMID: 31095288

[15] Ahola AJ Mutter S Forsblom C Harjutsalo V Groop P-H. Meal timing, meal frequency, and breakfast skipping in adult individuals with type 1 diabetes – associations with glycaemic control. Sci Rep. (2019) 9:20063. doi: 10.1038/s41598-019-56541-5, PMID: 31882789 PMC6934661

[16] Smith KJ Gall SL McNaughton SA Cleland VJ Otahal P Dwyer T. Lifestyle behaviours associated with 5-year weight gain in a prospective cohort of Australian adults aged 26-36 years at baseline. BMC Public Health. (2017) 17:54. doi: 10.1186/s12889-016-3931-y, PMID: 28068968 PMC5223543

[17] Gwin JA Leidy HJ. A review of the evidence surrounding the effects of breakfast consumption on mechanisms of weight management. Adv Nutr. (2018) 9:717–25. doi: 10.1093/advances/nmy047, PMID: 30204837 PMC6247188

[18] Pereira MA Erickson E McKee P Schrankler K Raatz SK Lytle LA. Breakfast frequency and quality may affect glycemia and appetite in adults and children. J Nutr. (2011) 141:163–8. doi: 10.3945/jn.109.114405, PMID: 21123469 PMC3001239

[19] Kito K Kuriyama A Takahashi Y Nakayama T. Impacts of skipping breakfast and late dinner on the incidence of being overweight: a 3-year retrospective cohort study of men aged 20–49 years. J Hum Nutr Diet. (2019) 32:349–55. doi: 10.1111/jhn.12640, PMID: 30821869

[20] Veasey R Haskell-Ramsay C Kennedy D Tiplady B Stevenson E. The effect of breakfast prior to morning exercise on cognitive performance, mood and appetite later in the day in habitually active women. Nutrients. (2015) 7:5712–32. doi: 10.3390/nu7075250, PMID: 26184302 PMC4517027

[21] Saintila J Calizaya-Milla YE Calizaya-Milla SE Elejabo-Pacheco AA Sandoval-Valentin GA Rodriguez-Panta SG. Association between nutritional knowledge, dietary regimen, and excess body weight in primary school teachers. J Multidiscip Healthc. (2022) 15:2331–9. doi: 10.2147/JMDH.S385713, PMID: 36267850 PMC9578462

[22] Cena H Calder PC. Defining a healthy diet: evidence for the role of contemporary dietary patterns in health and disease. Nutrients. (2020) 12:334. doi: 10.3390/nu12020334, PMID: 32012681 PMC7071223

[23] Schwingshackl L Morze J Hoffmann G. Mediterranean diet and health status: active ingredients and pharmacological mechanisms. Br J Pharmacol. (2020) 177:1241–57. doi: 10.1111/bph.14778, PMID: 31243760 PMC7056467

[24] Otzen T Manterola C. Sampling techniques on a population study. Int J Morphol. (2017) 35:227–32. doi: 10.4067/S0717-95022017000100037

[25] de Salud Ministerio del Perú Gobierno. Guía técnica para la valoración nutricional antropométrica de la persona adulta [Internet]. Lima. (2012). Available at: https://repositorio.ins.gob.pe/xmlui/handle/INS/225 (Accessed June 24, 2020).

[26] Vancini RL de Lira CAB Anceschi SA Rosa AV Lima-Leopoldo AP Leopoldo AS. Anxiety, depression symptoms, and physical activity levels of eutrophic and excess-weight brazilian elite police officers: a preliminary study. Psychol Res Behav Manag. (2018) 11:589–95. doi: 10.2147/PRBM.S186128, PMID: 30532604 PMC6241688

[27] Zurita S. Procedimientos de Laboratorio [Internet]. Instituto Nacional de Salud (2013) 240. Available at: http://bvs.minsa.gob.pe/local/minsa/2660.pdf

[28] Rodríguez-Sureda V Peinado-Onsurbe J. A procedure for measuring triacylglyceride and cholesterol content using a small amount of tissue. Anal Biochem. (2005) 343:277–82. doi: 10.1016/j.ab.2005.05.009, PMID: 15993372

[29] Sandoval Vegas MH Barrón Pastor HJ Loli Ponce RA Salazar Criado YV. Precisión en la determinación de glucosa, colesterol y trigliceridos séricos, en laboratorios clínicos de Lima, Perú. An la Fac Med. (2012) 73:233–8. doi: 10.15381/anales.v73i3.870

[30] MINSA. Guía técnica. Guía de Práctica Clínica para el diagnóstico, tratamiento y control de la diabetes mellitus tipo 2, en el primer nivel de atención. Perú: MINSA (2016).

[31] Huang C Niu K Momma H Kobayashi Y Guan L Chujo M. Breakfast consumption frequency is associated with grip strength in a population of healthy Japanese adults. Nutr Metab Cardiovasc Dis. (2014) 24:648–55. doi: 10.1016/j.numecd.2013.12.013 24598601

[32] Clayton DJ James LJ. The effect of breakfast on appetite regulation, energy balance and exercise performance. Proc Nutr Soc. (2016) 75:319–27. 26653842 10.1017/S0029665115004243

[33] Gwin JA Leidy HJ. Breakfast consumption augments appetite, eating behavior, and exploratory markers of sleep quality compared with skipping breakfast in healthy young adults. Curr Dev Nutr. (2018) 2:nzy074. doi: 10.1093/cdn/nzy074 30402594 PMC6215927

[34] Leidy HJ Hoertel HA Douglas SM Higgins KA Shafer RS. A high-protein breakfast prevents body fat gain, through reductions in daily intake and hunger, in “breakfast skipping” adolescents. Obesity. (2015) 23:1761–4. doi: 10.1002/oby.21185, PMID: 26239831

[35] Drewnowski A Rehm CD Vieux F. Breakfast in the United States: food and nutrient intakes in relation to diet quality in national health and examination survey 2011–2014. A study from the international breakfast research initiative. Nutrients. (2018) 10:1200. doi: 10.3390/nu10091200, PMID: 30200424 PMC6163505

[36] Aznar LAM Carou MDCV Sobaler AML Moreiras GV Villares JMM. Role of breakfast and its quality in the health of children and adolescents in Spain. Nutr Hosp. (2021) 38:396–409. doi: 10.20960/nh.03398 33724048

[37] Blasetti A Franchini S Castorani V Comegna L Fornari E Daniele F. Skipping breakfast is associated with an atherogenic lipid profile in overweight and obese prepubertal children. Int J Endocrinol. (2020) 2020:1–6. doi: 10.1155/2020/1849274, PMID: 33101407 PMC7569459

[38] Smith KJ Gall SL McNaughton SA Blizzard L Dwyer T Venn AJ. Skipping breakfast: longitudinal associations with cardiometabolic risk factors in the childhood determinants of adult health study. Am J Clin Nutr. (2010) 92:1316–25. doi: 10.3945/ajcn.2010.30101, PMID: 20926520

[39] de Souza MR Neves MEA Souza AM Muraro AP Pereira RA Ferreira MG. Skipping breakfast is associated with the presence of cardiometabolic risk factors in adolescents: study of cardiovascular risks in adolescents – ERICA. Br J Nutr. (2021) 126:276–84. doi: 10.1017/S0007114520003992, PMID: 33040745

[40] Moreno LA Rodríguez G Guillén J Rabanaque MJ León JF Ariño A. Anthropometric measurements in both sides of the body in the assessment of nutritional status in prepubertal children. Eur J Clin Nutr. (2002) 56:1208–15. doi: 10.1038/sj.ejcn.1601493, PMID: 12494306

[41] Li Q Wu C Ma P Cui H Li R Hong C. Breakfast consumption frequency is associated with dyslipidemia: a retrospective cohort study of a working population. Lipids Health Dis. (2022) 21:33. doi: 10.1186/s12944-022-01641-x, PMID: 35351127 PMC8966363

[42] Ofori-Asenso R Owen AJ Liew D. Skipping breakfast and the risk of cardiovascular disease and death: a systematic review of prospective cohort studies in primary prevention settings. J Cardiovasc Dev Dis. (2019) 6:30. doi: 10.3390/jcdd6030030, PMID: 31443394 PMC6787634

